# A novel discrete zeroing neural network for online solving time-varying nonlinear optimization problems

**DOI:** 10.3389/fnbot.2024.1446508

**Published:** 2024-08-06

**Authors:** Feifan Song, Yanpeng Zhou, Changxian Xu, Zhongbo Sun

**Affiliations:** ^1^School of Finance, Changchun Finance College, Changchun, China; ^2^VanJee Technology Co., Ltd., Beijing, China; ^3^Department of Mechanical and Electrical Engineering, Changchun University of Technology, Changchun, China; ^4^Department of Control Engineering, Changchun University of Technology, Changchun, China

**Keywords:** path planning, discrete zeroing neural network, time-varying nonlinear optimization problem, 0-stability, real-time capability

## Abstract

To reduce transportation time, a discrete zeroing neural network (DZNN) method is proposed to solve the shortest path planning problem with a single starting point and a single target point. The shortest path planning problem is reformulated as an optimization problem, and a discrete nonlinear function related to the energy function is established so that the lowest-energy state corresponds to the optimal path solution. Theoretical analyzes demonstrate that the discrete ZNN model (DZNNM) exhibits zero stability, effectiveness, and real-time performance in handling time-varying nonlinear optimization problems (TVNOPs). Simulations with various parameters confirm the efficiency and real-time performance of the developed DZNNM for TVNOPs, indicating its suitability and superiority for solving the shortest path planning problem in real time.

## 1 Introduction

In recent years, the application of mobile platforms has been increasing, enhancing the efficiency of production systems (Balk et al., 2021; Wu et al., 2022; Zhou et al., 2022). In this case, certain collisions can delay production, harm productivity, and reduce profits (Gonzalez et al., 2016; Li et al., 2022). Therefore, the path planning problem for mobile platforms has become a research hotspot. In the processes of handling, loading, and unloading, the path planning problem for the mobile platform can be transformed into a shortest path planning problem, thereby saving both time and cost. Therefore, the shortest path problem is a typical combinatorial optimization problem that seeks the shortest path from a specified starting point to a desired terminus, aiming to minimize the total path cost (Zhang and Li, 2017; Li et al., 2021; Xu et al., 2022).

Path-planning methods are classified as follows: The artificial potential field method is a virtual force approach based on physical design (Jie et al., 2017; Zhou et al., 2023a,b). In this algorithm, movement toward the target point is likened to gravitation, while movement away from obstacles is likened to repulsion. Thus, the path planning problem is transformed into an optimization problem using a gravitational repulsion field function (Robinson et al., 2020). The model is simple to establish but challenging to obtain the optimal solution due to its tendency to converge to local optima. The fuzzy logic algorithm, derived from fuzzy control, emulates path-seeking methods based on drivers' daily driving experiences. It directly utilizes expert knowledge from a database, offering good stability when incorporating real-time external information. However, the effectiveness of the fuzzy rules in the expert database relies heavily on accumulated experience, and the algorithm may lack real-time responsiveness in rapidly changing external environments. Graph search-based methods include the D* algorithm (Raheem and Ibrahim, 2018) and the Lee algorithm (Chi et al., 2022), etc. One of the most representative algorithms is the greedy algorithm, which aims to find the target point. In order to accelerate the optimization speed and avoid constraints, Fu improved the A* algorithm on the basis of the greedy algorithm to solve the path planning problem of industrial mobile manipulators. Under safe and non-collision conditions, a local path optimization strategy is directly adopted to reduce the number and length of local paths by straightening local paths (Fong et al., 2016; Fu et al., 2018). However, the algorithm lacks real-time performance due to the extensive computational requirements of the planning problems involving high-dimensional mobile platforms and snake-like robots. Bionic algorithms are developed for such problems, such as the genetic algorithm (Yang et al., 2008), the neural network algorithm (Qiu et al., 2018; Buddhadeb et al., 2020; Wang et al., 2022), and the ant colony algorithm (Song et al., 2021). The ant colony algorithm achieves optimization by simulating the foraging behavior of ant colonies, offering advantages such as parallelism and global optimization. Nevertheless, it is easy to fall into the local optimal solution due to the large number of calculations. Hui proposes an ant colony optimization algorithm to create a collapse-free incipient path in the intricate map and then applies a turning point optimization algorithm to achieve path planning on the mobile platform (Yang et al., 2019). Xu uses a particle swarm optimization algorithm to create a linear path and then smooths the linear path. However, vibrations can arise at the intersections of each path, potentially causing the anticipated trajectory to lose its optimality. This may result in the mobile platform stopping, rotating, and then restarting (Xu et al., 2021; Song et al., 2022). Therefore, a higher-order Bezier curve is utilized to construct the desired path directly to overcome the above problems. Nevertheless, it is necessary to design an efficient algorithm with strong computing power that is less time-consuming to find the optimal path in an environment with a large scanning area and a large number of obstacles. Hence, the above algorithms are always limited by the inherent problem of the exponential growth of the search scale. As the number of nodes increases, the success rate of solving within a limited time is significantly weakened. Neural networks are powerful algorithms to solve many scientific research and engineering problems. For the shortest path planning problem, Song proposed a pulse-coupled neural network model with a special mechanism to solve the shortest path planning problem. Compared with numerous algorithms, it effectively reduces costs (Sang et al., 2016). Filipe proposes a two-layer Hopfield neural network to solve the shortest path, which requires fewer neurons and converges quickly. However, the solution of this model is not optimal when dealing with shortest path planning problems (Araujo et al., 2001). In general, the discrete zeroing neural network (DZNN) has the characteristics of parallel processing, which can be used in path planning to quickly solve the optimal path and achieve the path planning task of the mobile platform (Hopfield and Tank, 1985).

The rest of this paper covers the following four parts: Section 2 describes the mathematical models of path planning and the ZNN model (DZNNM). Section 3 provides a theoretical analysis of the stability and convergence of the proposed DZNNM. In Section 4, the superiority and real-time characteristics of the proposed DZNNM are verified by numerical simulations. Section 5 draws the conclusion and future works. The primary contributions of this paper are described as follows:

1) The path planning problem is converted to the nonlinear optimization problem with equality and inequality constraints, and the nonlinear function related to the energy function is constructed so that the solution of the lowest energy state corresponds to the solution under the optimal path.
2) The theoretical analysis shows that the proposed discrete ZNN method has 0-stability and convergence for time-varying nonlinear optimization problems (TVNOPs).
3) The simulation results demonstrate that the DZNNM is feasible, effective, and real-time in dealing with the shortest path planning problem.

## 2 Problem formulation and model foundation

In this section, it describes the process of transforming the path planning problem into a nonlinear optimization problem. It covers the conversion process from the continuous ZNN model to the DZNNM and the establishment of the mathematical model for path planning. Specifically, it presents the problem formulation, the ZNN model, and the energy function model for online solving of TVNOPs.

### 2.1 Problem formulation

Let *L* = {*j*|*j* = 1, ⋯*m*} denote an arbitrary finite set, and let *B* = (*j, r*), (*j* ∈ *L, r* ∈ *L*) represent a set of ordered pairs of elements arranged sequentially. (*j, r*) and (*r, j*) represent different elements if and only if *r* is equal to *j*. *T* = (*L, D*) is an oriented graph, and *D* ⊂ *B*. The parameters of *L* are named vertexes, and the parameters of *D* are denoted as oriented borders. If a cost matrix *c*_*jr*_ corresponds to edges in *T* from vertex *j* to vertex *r*, then *T* is referred to as a directed graph. Generally, the cost matrix *c*_*jr*_ is not needfully symmetric. In other words, the cost from vertex *r* to *j* is not inequivalent, possibly to the cost from vertex *j* to *r*. In addition, some borders between vertexes do not exist. Namely, the number of borders may be less than the quantity of vertices. For non-existent edges, the value of the cost coefficient is defined as infinity. In this paper, the shortest path problem is to search for the shortest feasible path from the desired starting point to the designated terminus.

### 2.2 Mathematical model of path planning

Consider the shortest path from vertex 1 to vertex *m* for an oriented graph with *m* vertexes and *m* borders, and the price of *c*_*jr*_ per border. To formulate the shortest path problem, there are two typical path representations: vertex representation and edge representation. This paper adopts the border path characterization method to express the shortest path problem. The shortest path problem can be transformed into the following integer linear programming problem (Xia and Wang, 2000; Yoshihiko and Willsky, 2015).


(1) $$\begin{array}{l} \begin{array}{l} \text{min}\overset{m}{\underset{j=1}{\sum }}\overset{m}{\underset{r=1}{\sum }}c_{jr}x_{jr}\\ \text{s}.\text{t}.\overset{m}{\underset{v=1}{\sum }}x_{jv}\;-\;\overset{m}{\underset{l=1}{\sum }}x_{lj}=\{\begin{array}{l} 1,\text{if }j=1\\ 0,\text{if }j=2,3\cdots m-1\\ -1,\text{if }j=m \end{array} \end{array}, \end{array}$$

where the minimizing objective function of an integer linear programming problem (Equation 1) is the absolute price of the route, and the restriction is -1, 0, or 1. The constraint guarantees a sequential route from the specified starting point to the particular ending point. A decision variable represented by edge dependence from vertex to vertex is defined as follows:


(2) $$\begin{array}{l} x_{jr}=\{\begin{array}{l} 1,\text{if the edge from}j\text{to}r\text{ is on the path },\\ 0,\text{otherwise} \end{array} \end{array}$$

Due to the constraint coefficient matrix defined in Equation 2, it is set to either 0 or 1. If there exists a unique optimal integer solution where the variable takes on values of 0 or 1, the integer programming mentioned above can be transformed into the following linear programming (Equation 3):


(3) $$\begin{array}{l} \begin{array}{l} \text{min}\overset{m}{\underset{j=1}{\sum }}\overset{m}{\underset{r=1}{\sum }}c_{jr}x_{jr}\\ \text{s. t.}\overset{m}{\underset{v=1}{\sum }}x_{jv}-\overset{m}{\underset{l=1}{\sum }}x_{lj}=\delta_{j1}\;-\;\delta_{jm}\\ \;x_{jr}\geq 0,\;j,r=1,2,\cdots m \end{array}, \end{array}$$

where δ_*jr*_ denotes the Kronecker function, which is defined as *j* = *r*, δ_*jr*_ = 1, and *j* ≠ *r*, δ_*jr*_ = 0. According to the duality principle of convex optimization (Lemeshko and Sterin, 2013), the dual path planning problem (Equation 4) can be obtained as follows:


(4) $$\begin{array}{l} \begin{array}{l} \text{max }y_{1}-y_{m}\\ \text{s. t. }y_{j}-y_{r}\leq c_{jr}\;j,r=1,2\cdots m \end{array}, \end{array}$$

where *y*_*j*_ represents the dual decision variable related to vertex *j*, *y*_1_ − *y*_*j*_ indicates the shortest length from vertex 1 to vertex *j*.

Generally, a suitable energy function is developed such that the lowest energy condition corresponds to the anticipative solution. According to the duality properties of linear programming, an energy function model (Equation 5) of the original duality problem is generalized by Xia and Wang (2000):


(5) $$\begin{array}{l} E\left(x,y\right)=\frac{1}{2}\overset{m}{\underset{j=1}{\sum }}{\left[\overset{m}{\underset{r=1}{\sum }}\left(x_{jr}-x_{rj}\right)-\delta_{i1}+\delta_{im}\right]}^{2}\\ +\frac{1}{2}\overset{m}{\underset{j=1}{\sum }}\overset{m}{\underset{r=1}{\sum }}{\left[{\left(-x_{jr}\right)}^{+}\right]}^{2}\\ +\frac{1}{2}{\left[\overset{m}{\underset{j=1}{\sum }}\overset{m}{\underset{r=1}{\sum }}c_{jr}x_{jr}-y_{1}+y_{m}\right]}^{2}\\ +\frac{1}{2}\overset{m}{\underset{j=1}{\sum }}\overset{m}{\underset{r=1}{\sum }}{\left[{\left(y_{j}-y_{r}-c_{jr}\right)}^{+}\right]}^{2}, \end{array}$$

where (*s*)^+^ = max{0, *s*}, and *s* ∈ *R*. The first term of the above formula represents the equality constraint, the second term denotes the non-negative constraint, the third term means the square dual gap, and the last term indicates the inequality restriction in the dual problem.

For convenience, the following coefficient vectors are defined as:


$$\begin{array}{l} ŷ={\left({ŷ}_{1},\cdots \;,{ŷ}_{m}\right)}^{⊤};\\ ĉ={\left({ĉ}_{11},\cdots \;,{ĉ}_{1m},{ĉ}_{21},\cdots \;,{ĉ}_{2m},\cdots \;,{ĉ}_{m1},\cdots \;,{ĉ}_{mm}\right)}^{⊤};\\ x={\left(x_{11},\cdots \;,x_{1m},x_{21},\cdots \;,x_{2m},\cdots \;,x_{m1},\cdots \;,x_{mm}\right)}^{⊤}. \end{array}$$

Define *A* is an *m* × *m*^2^ constraint matrix, whose row denotes *j* and column means *r*, *e*_*j*_ − *e*_*r*_ is a vector, the *j* element is 1, and the other elements are 0.

The above formula can be rewritten as Equation 6:


(6) $$\begin{array}{l} E\left(x,ŷ\right)=\frac{1}{2}\left[{\left(c^{⊤}x-{\left(e_{1}-e_{m}\right)}^{⊤}ŷ\right)}^{2}+||{\left(-x\right)}^{+}|{|}_{2}^{2}+||{\left(A^{⊤}ŷ-c\right)}^{+}|{|}_{2}^{2}+||Ax+e_{m}-e_{1}|{|}_{2}^{2}\right]. \end{array}$$

Let $\tilde{b}=e_{1}-e_{m}$. Therefore, the above equation can be simplified as Equation 7:


(7) $$\begin{array}{l} E\left(x,ŷ\right)=\frac{1}{2}\left[{\left(c^{⊤}x-{\tilde{b}}^{⊤}ŷ\right)}^{2}+||{\left(-x\right)}^{+}|{|}_{2}^{2}+||{\left(A^{⊤}ŷ-c\right)}^{+}|{|}_{2}^{2}+||Ax-\tilde{b}|{|}_{2}^{2}\right], \end{array}$$

where ‖·‖ represents the 2-norm, and given ${\left(-x\right)}^{+}={\left[{\left(-x_{1}\right)}^{+},\cdots \;,{\left(-x_{m}\right)}^{+}\right]}^{⊤}$.

### 2.3 Continuous ZNN model and discrete ZNN model

Through the above analysis, the path planning problem is transformed into the path optimization problem from the specified initiating point to the terminus. An energy function is established for the shortest path to solve the optimization problem. The state solution corresponding to the optimal node is obtained when the energy function reaches its minimum. Therefore, the shortest path problem is considered as the TVNOP. The TVNOPs described in discrete time form are as follows (Guo et al., 2017; Qiu et al., 2018; Sun et al., 2021):


(8) $$\begin{array}{l} \min\hat{f}\left(x^{\chi +1},t_{\chi +1}\right),\left[t_{\chi },t_{\chi +1}\right)\in \left[0,+\infty \right), \end{array}$$

where $\hat{f}\left(\cdot ,\cdot \right)$ represents a differentiable nonlinear function (Equation 8). The discrete form of the TVNOPs is transformed from the continuous time-varying nonlinear function $\hat{f}\left(x\left(t\right),t\right)$ based on the sampling time *t* = (χ + 1)τ. τ > 0 is the acquisition interval, and χ = 0, 1, 2, ⋯  is the sampling time. The existing and foregone data is used to ensure the next data iteratively, which can solve TVNOPs. In the calculation time interval [*t*_χ_, *t*_χ+1_) ∈ [0, +∞), the variable *x*_χ+1_ and the function $\hat{f}\left(x^{\chi +1},t_{\chi +1}\right)$ can be calculated iteratively by given information *x*_χ_ and $\hat{f}\left(x^{\chi },t_{\chi }\right)$ at the next moment.

A DZNNM is acquired for solving TVNOPs online; the following continuous TVNOPs (Equation 9) are considered:


(9) $$\begin{array}{l} \underset{x\left(t\right)\in R^{n}}{\min}\hat{f}\left(x\left(t\right),t\right)\in R,t\in \left[0,+\infty \right). \end{array}$$

On behalf of solving the time-varying optimal solution of continuous TVNOPs x^*^(*t*), the gradient of the function $\hat{f}\left(x\left(t\right),t\right)$ (Equation 10) is directly generalized as:


(10) $$\begin{array}{l} \vartheta \left(x\left(t\right),t\right)=\partial \hat{f}\left(x\left(t\right),t\right)/\partial x\left(t\right). \end{array}$$

The above formula is expanded to the following Equation 11:


(11) $$\begin{array}{l} {\left[\frac{\partial \hat{f}}{\partial x_{1}},\frac{\partial \hat{f}}{\partial x_{2}},\cdots \frac{\partial \hat{f}}{\partial x_{n}}\right]}^{⊤}=[\vartheta_{1}\left(x\left(t\right),t\right),\vartheta_{2}\left(x\left(t\right),t\right),\cdots ,\\ \;{\vartheta_{n}\left(x\left(t\right),t\right)]}^{⊤}\in R^{n}, \end{array}$$

where the superscript ⊤ represents the transposition operational character of a matrix or a vector. The gradient ϑ(*x*(*t*), *t*) is a slippy differentiable nonlinear function created by the objective function $\hat{f}\left(x\left(t\right),t\right)$. On behalf of solving the theoretical solution of TVNOPs, the gradient of the objective function tends to 0, and the zeroing dynamical system (Equation 12) is defined as:


(12) $$\begin{array}{l} {\dot{\vartheta }}_{t}\left(x\left(t\right),t\right)=\frac{\text{d}\vartheta \left(x\left(t\right),t\right)}{\text{d}t}=-\lambda \vartheta \left(x\left(t\right),t\right), \end{array}$$

where the parameter λ > 0, ${\dot{\vartheta }}_{t}\left(x\left(t\right),t\right)$ is the derivative of the gradient ϑ(*x*(*t*), *t*) in connection with time. While the error ϑ(*x*(*t*), *t*) reaches 0, the solution *x*(*t*) of the TVNOPs arrives at the corresponding theoretical solution x^*^(*t*) of the continuous TVNOPs (Sun et al., 2020a,b; Wei et al., 2021). Because of the zeroing dynamic system (Equation 12), the differential equation of the ZNN model (Equation 13) is extended as:


(13) $$\begin{array}{l} \tilde{H}\left(x\left(t\right),t\right)ẋ\left(t\right)=-\lambda \vartheta \left(x\left(t\right),t\right)-{\dot{\vartheta }}_{t}\left(x\left(t\right),t\right), \end{array}$$

where $\tilde{H}\left(x\left(t\right),t\right)$ is a non-singular Hessian matrix. The details can be seen as follows:


$$\begin{array}{l} \tilde{H}\left(x\left(t\right),t\right)=\frac{\partial^{2}\hat{f}\left(x\left(t\right),t\right)}{\partial x\left(t\right)\partial x^{⊤}\left(t\right)}\\ =\left[\begin{array}{cccc} \frac{\partial^{2}\hat{f}\left(x\left(t\right),t\right)}{\partial x_{1}\partial x_{1}} & \frac{\partial^{2}\hat{f}\left(x\left(t\right),t\right)}{\partial x_{1}\partial x_{2}} & \cdots & \frac{\partial^{2}\hat{f}\left(x\left(t\right),t\right)}{\partial x_{1}\partial x_{n}}\\ \frac{\partial^{2}\hat{f}\left(x\left(t\right),t\right)}{\partial x_{2}\partial x_{1}} & \frac{\partial^{2}\hat{f}\left(x\left(t\right),t\right)}{\partial x_{2}\partial x_{2}} & \cdots & \frac{\partial^{2}\hat{f}\left(x\left(t\right),t\right)}{\partial x_{2}\partial x_{n}}\\ \vdots & \vdots & \ddots & \vdots \\ \frac{\partial^{2}\hat{f}\left(x\left(t\right),t\right)}{\partial x_{n}\partial x_{1}} & \frac{\partial^{2}\hat{f}\left(x\left(t\right),t\right)}{\partial x_{n}\partial x_{2}} & \cdots & \frac{\partial^{2}\hat{f}\left(x\left(t\right),t\right)}{\partial x_{n}\partial x_{n}} \end{array}\right]\in R^{n\times n}. \end{array}$$

Due to the non-singularity of the Hessian matrix, the above equation is converted to the following Equation 14:


(14) $$\begin{array}{l} ẋ\left(t\right)=-{\tilde{H}}^{-1}\left(x\left(t\right),t\right)\left(\lambda \vartheta \left(x\left(t\right),t\right)+{\dot{\vartheta }}_{t}\left(x\left(t\right),t\right)\right). \end{array}$$

If the Hessian matrix is a positive symmetric matrix, it represents the solution of the continuous TVNOPs. Moreover, if the matrix is singular, the Hessian matrix can be transformed into $\tilde{H}+rI$, where *r* is the absolute value of the maximum eigenvalue of the Hessian matrix and *I* is the identity matrix. Thus, the matrix $\tilde{H}\left(x\left(t\right),t\right)$ satisfies the non-singularity condition.

A DZNNM is proposed to solve the TVNOPs to solve the optimal value of the energy function *E*(*x*, ŷ). Hence, a continuous ZNN model is discretized to obtain the ZNN model in discrete form. Generally, Euler's forward difference equation ẋ(*t*) = (*x*^χ+1^ − *x*^χ^)/τ is employed to discretize the continuous ZNN model (Equation 15) as follows:


(15) $$\begin{array}{l} x^{\chi +1}=x^{\chi }-{\tilde{H}}^{-1}\left(x^{\chi },t_{\chi }\right)\left(\iota \vartheta \left(x^{\chi },t_{\chi }\right)+\tau {\dot{\vartheta }}_{t}\left(x^{\chi },t_{\chi }\right)\right). \end{array}$$

The above formula can be called as DZNNM, where ι = τλ ∈ (0, 1] is step length, ${\tilde{H}}^{-1}\left(x^{\chi },t_{\chi }\right)$, ϑ(*x*_χ_, *t*_χ_), and ${\dot{\vartheta }}_{t}\left(x_{\chi },t_{\chi }\right)$ are discrete forms of ${\tilde{H}}^{-1}\left(x\left(t\right),t\right)$, ϑ(*x*(*t*), *t*), and ${\dot{\vartheta }}_{t}\left(x\left(t\right),t\right)$, respectively.

## 3 Theoretical analyzes and results

The continuous ZNN model construction process and the DZNNM construction process are briefly described to solve the TVNOPs in the previous section. Therefore, the continuous ZNN model-building process, discretization steps, and proof process are elaborated on in this section. Define a continuously differentiable linear equation, and the matrix *Q*(*t*) is a known and bounded matrix of time-varying full-rank coefficients; *w*(*t*) is a time-varying vector and is differentiable at any time in connection with time *t*.


(16) $$\begin{array}{l} \nu \left(x\left(t\right),t\right)=Q\left(t\right)x\left(t\right)-w\left(t\right)=0. \end{array}$$

The discrete formal equation corresponding to the above formula (Equation 16) can be managed in the time period [χτ, (χ + 1)τ] ⊆ [*t*_0_, *t*_*f*_]. The time-varying equation in discrete form Equation 17 is as follows:


(17) $$\begin{array}{l} Q_{\chi +1}x_{\chi +1}=w_{\chi +1}, \end{array}$$

where the matrices *Q*_χ+1_ and *w*_χ+1_ are discrete forms of the matrices *Q*(*t*) and *w*(*t*), respectively. Instantaneous sampling is *t* = (χ + 1)τ, i.e., χ = 0, 1, 2⋯  denotes the regenerative target.

The following vector-valued error function ν(*x*(*t*), *t*) = *Q*(*t*)*x*(*t*)−*w*(*t*) is defined to handle the above equation based on the design steps by Zhang et al. (2015). The continuous ZNN model (Equation 18) of the linear equation dynamical system has the following form:


(18) $$\begin{array}{l} ẋ\left(t\right)=Q^{-1}\left(t\right)\left(ẇ\left(t\right)-\dot{Q}\left(t\right)x\left(t\right)-\eta \left(Q\left(t\right)x\left(t\right)-w\left(t\right)\right)\right). \end{array}$$

Among them is the design parameter η > 0, which can be used to control the convergent ratio. *Q*^−1^(*t*) denotes inverse and it is equivalent to *H*^−1^(*t*). Generally, the ZNN model is discretized by Euler's forward difference formula (Equation 19) as follows:


(19) $$\begin{array}{l} x_{\chi +1}=x_{\chi }+Q_{\chi }^{-1}\left(\tau {ẇ}_{\chi }-\tau {\dot{Q}}_{\chi }x_{\chi }-h\left(Q_{\chi }x_{\chi }-w_{\chi }\right)\right). \end{array}$$

**Definition 1** (Guo and Zhang, 2012; Jin and Zhang, 2015; Zhang et al., 2015). The roots of the characteristic polynomial $P_{M}\left(\psi \right)=\overset{M}{\underset{i=0}{\sum }}\omega_{i}\psi^{i}$ are used to verify whether the *M*-step method $\overset{M}{\underset{i=0}{\sum }}\omega_{i}\sigma_{\chi +i}=\tau \overset{M}{\underset{i=0}{\sum }}\zeta_{i}\varpi_{\chi +i}$ has 0-stability .

The *M*-step method has 0-stability if the solution of the equation *p*_*M*_(ψ) = 0 lies on or within the unit circle (i.e., |ψ| ≤ 1). The convergence order *O*(τ^*p*^) of the *M*-step method matches the truncation error order *p* (*p* > 0) of the equation solution.

**Definition 2** (Jin and Zhang, 2015; Jin et al., 2018). If and only if *M*-step has 0-stability and is consistent over time *t* ∈ [*t*_0_, *t*_*f*_], it is convergent (i.e., $\sigma_{\left[\left(t-t_{0}\right)/\tau \right]}\to \sigma^{*}\left(t\right)$ with τ → 0) .

**Definition 3**. The 0-stable consistency of the *M*-step method converges to the order of its truncation error.

Based on the aforementioned definitions, we will analyze the 0-stability and convergence performance of the DZNNM.

**Theorem 1**. The DZNNM is 0-stable.

**Proof**. A DZNNM (Equation 15) is viewed as the one-step neural network dynamics on account of Definition 1. According to Definition 1, the characteristic polynomial (Equation 20) of the ZNN model separated and dispersed by forward Euler interpolation is as follows:


(20) $$\begin{array}{l} P_{1}\left(\psi \right)=\psi -1. \end{array}$$

The root (Equation 21) of the above equation is


(21) $$\begin{array}{l} \psi_{1}=1. \end{array}$$

Therefore, according to Definition 1, the DZNNM is 0-stable. The proof is fulfilled.

**Theorem 2**. The DZNNM (Equation 15) converges to the order of the truncation error *O*(τ^2^).

**Proof**. The forward Euler interpolation formula (Equation 22) is as follows:


(22) $$\begin{array}{l} {ẋ}_{\chi }=\frac{x_{\chi +1}-x_{\chi }}{\tau }+O\left(\tau \right). \end{array}$$

The continuous ZNN model (Equation 18) is discretized by forward Euler interpolation, and the following formula (Equation 23) is obtained:


(23) $$\begin{array}{l} x_{\chi +1}=x_{\chi }+Q_{\chi }^{-1}\left(\tau {ẇ}_{\chi }-\tau {\dot{Q}}_{\chi }x_{\chi }-h\left(Q_{k}x_{\chi }-w_{\chi }\right)\right)+O\left(\tau^{2}\right). \end{array}$$

In the light of the above analysis, the truncation error of the DZNNM is *O*(τ^2^), so the DZNNM has consistency, convergence, and 0-stability. According to Definition 2 and Definition 3, the order of convergence of the model is *O*(τ^2^). The proof is fulfilled.

**Theorem 3**. For the TVNOPs in discrete form, the steady-state position error $\underset{\chi \to \infty }{\lim}{\left\|Q_{\chi }x_{\chi }-w_{\chi }\right\|}_{2}$ of the DZNNM has order *O*(τ^2^).

**Proof**. According to Theorem 1, Theorem 2, and Definition 3, as χ tends to infinity, we can get $x_{\chi }^{*}+O\left(\tau^{2}\right)=x_{\chi }$. Therefore, the following derivation process (Equation 24) is obtained:


(24) $$\begin{array}{l} ||Q_{\chi }x_{\chi }-w_{\chi }|{|}_{F}=||Q_{k}\left(x^{*}+O\left(\tau^{2}\right)\right)-w_{\chi }|{|}_{F}\\ =||Q_{\chi }x^{*}-w_{\chi }+Q_{\chi }O\left(\tau^{2})\right)|{|}_{F}, \end{array}$$

where ‖ ‖_*F*_ is a Fubini norm. The following Equation (25) is obtained by further arrangement:


(25) $$\begin{array}{l} ||Q_{\chi }x_{\chi }-w_{\chi }|{|}_{F}=||Q_{\chi }O\left(\tau^{2})\right)|{|}_{F}=O\left(\tau^{2}\right). \end{array}$$

This proof is thus completed.

## 4 Numerical simulations and verifications

**Figure 1:**
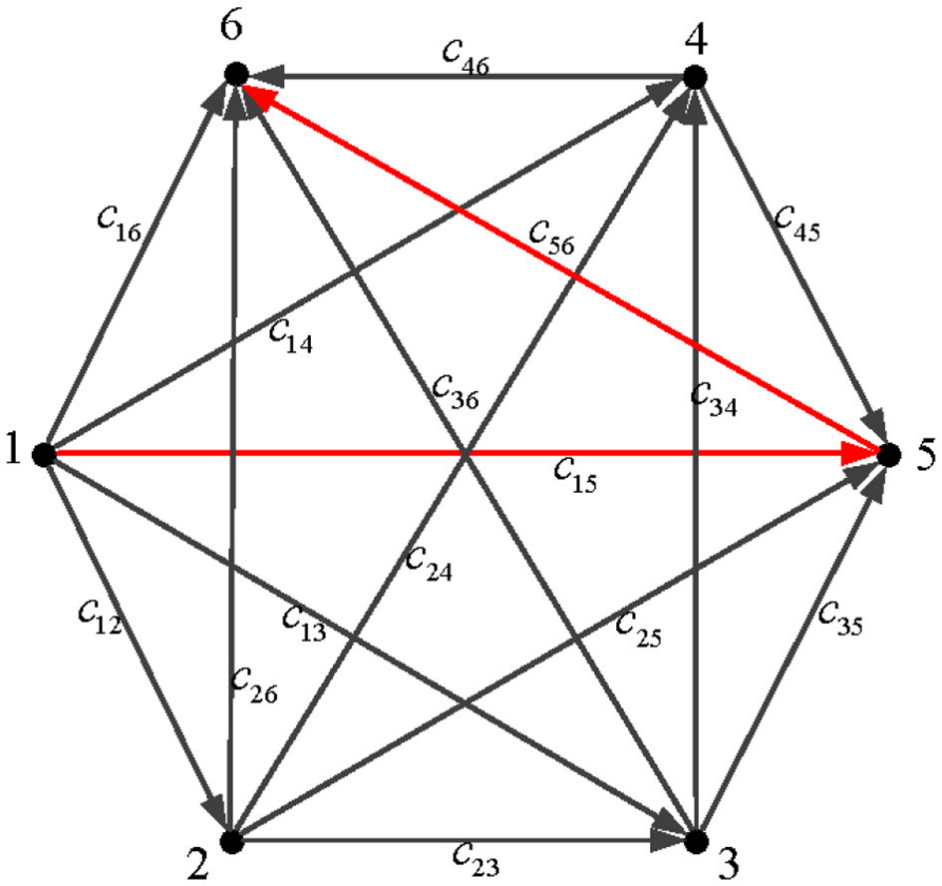
The transport diagram of six-node path planning.

Consider the shortest path planning problem, where each node has five possible directions to move from a fixed initial point to the terminus. To make the transportation process more reasonable, the following conditions are assumed to be true:

1) For the path planning problem with a single starting point and a single target point, node 1 is the starting point and node 6 is the endpoint, as shown in Figure 1.
2) In order to meet the actual transportation situation, some transportation roads do not exist. For example, it cannot travel from node 4 to node 4. Therefore, the given value of the cost *c*_*jr*_ of such a path is large in the simulation.
3) In the transportation process, it should not go in the reverse direction. For instance, there is no arrow to go from node 2 to node 1, indicating that the situation is not considered.
4) Assume that each node can go to a node whose number is greater than its own.

### 4.1 Six-node path planning simulations

The path planning problem is to find the shortest path between source node 1 and terminal node 6, so as to minimize the cost *E*(*x, y*) in the transportation process. The cost coefficient matrix ĉ needs to be set in the process of establishing the path planning mathematical model in Section 2.2, which is given in Table 1. The cost coefficient matrix is $c={\left[c_{11},c_{12},\cdots c_{16},\cdots \;,c_{61},\cdots c_{66}\right]}^{⊤}$, and the original value matrix of the system is defined as follows: $x\left(0\right)={\left[0,0,\cdots 0\right]}_{36*1}^{⊤}$, $y\left(0\right)={\left[1,1,\cdots 1\right]}_{6*1}^{⊤}$. In Section 2.4, it is noted that different parameters of the DZNNM generally exhibit different convergence rates. Therefore, the parameters are set as ι = 0.1, ι = 0.5, ι = 0.9, and ι = 1.0, respectively. The energy function of each path is calculated successively to determine the shortest path in the transportation process.

**Table 1:** Cost coefficients of six-node path planning problems.

	***c*_1_**	***c*_2_**	***c*_3_**	***c*_4_**	***c*_5_**	***c*_6_**
*c*_1_	100	16	8	16	9	8
*c*_2_	100	100	9	10	11	9
*c*_3_	100	100	100	10	11	12
*c*_4_	100	100	100	100	9	10
*c*_5_	100	100	100	100	100	13
*c*_6_	100	100	100	100	100	100

The simulation results show that the DZNNM is exploited to solve the shortest path planning problem. As the number of iterations increases, the energy function *E*(*x, y*) decreases to 0, indicating that the DZNNM can effectively address the path planning problem with a single starting point and a single target point. While the energy function gets its minimum value, the optimal solution $\hat{x}$ can be solved at this time. The optimal solution $\hat{x}$ is substituted into the energy function *E*(*x, y*) to obtain the cost of each path so as to determine the shortest path. Starting from node 1, it uses the energy function to calculate the energy consumption from node 1 to node 2, node 3, node 4, node 5, and node 6. The specific energy loss is shown in the Tables 2, 3.

**Table 2:** Energy loss from node 1 to other nodes.

**|*E*_12_|**	**|*E*_13_|**	**|*E*_14_|**	**|*E*_15_|**	**|*E*_16_|**
5.5558	2.1937	1.6309	1.3792	2.5478

**Table 3:** Energy loss from node 5 to other nodes.

**|*E*_51_|**	**|*E*_52_|**	**|*E*_53_|**	**|*E*_54_|**	**|*E*_56_|**
15.3249	20.9741	8.3363	3.4891	2.9722

**Figure 2:**
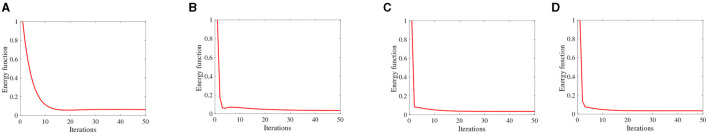
Energy function based on the six-node primal duality problem, **(A)** ι = 0.1, **(B)** ι = 0.5, **(C)** ι = 0.9, and **(D)** ι = 1.0.

As can be seen in Table 2, it can be concluded that the cost from node 1 to node 5 is the smallest. Therefore, the energy consumption of node 5 compared to other nodes is calculated. According to the analysis of the actual transportation situation and assumed conditions, when the mobile platform moves to node 5, it can only transport objects to target point 6. In order to verify the effectiveness of the algorithm, the optimal solution is substituted into the expression of the energy function to solve the energy loss from node 5 to each node. Table 3 can testify to the validity of the DZNNM. Therefore, the shortest path is from node 1 to node 5, and finally to target point 6. Figure 2, Tables 2, 3 indicate that the DZNNM is effective in processing TVNOPs. As the number of iterations increases, the energy function decreases to 0 in a short number of times, which reflects the high efficiency and real-time performance of the DZNNM.

**Figure 3:**
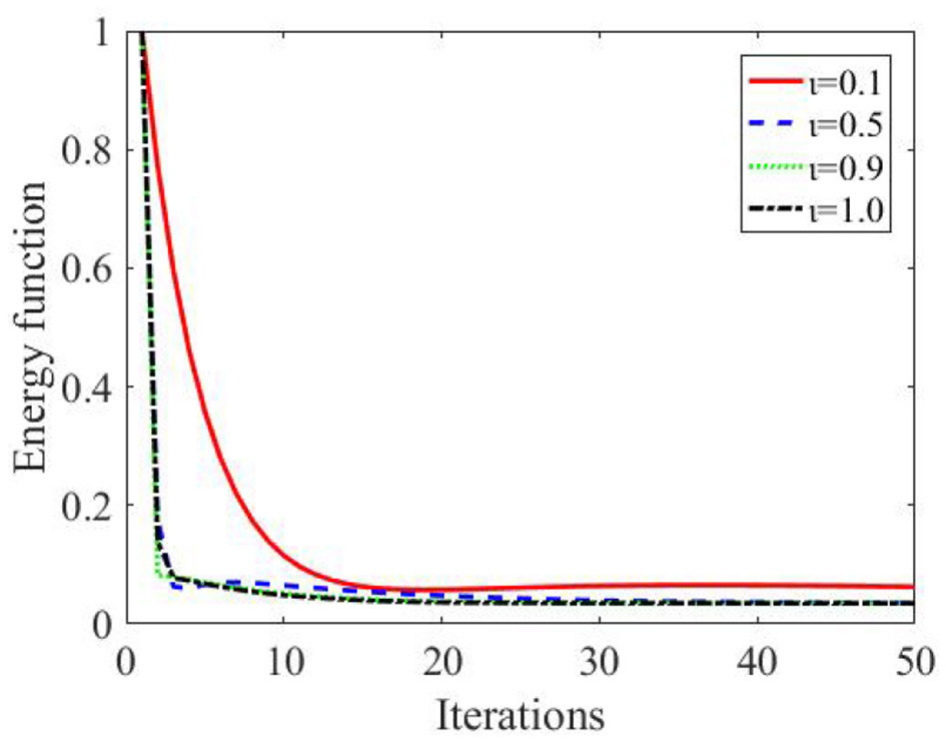
Energy loss under different parameters.

As the parameter ι increases, the energy function rapidly converges to 0, reflecting the fast convergence and effectiveness of the DZNNM, as shown in Figure 3. In practical application, adjusting parameters can accelerate the convergence rate of the whole optimal path, which can quickly accelerate and complete the path planning.

### 4.2 Path planning simulations of 12 nodes

**Figure 4:**
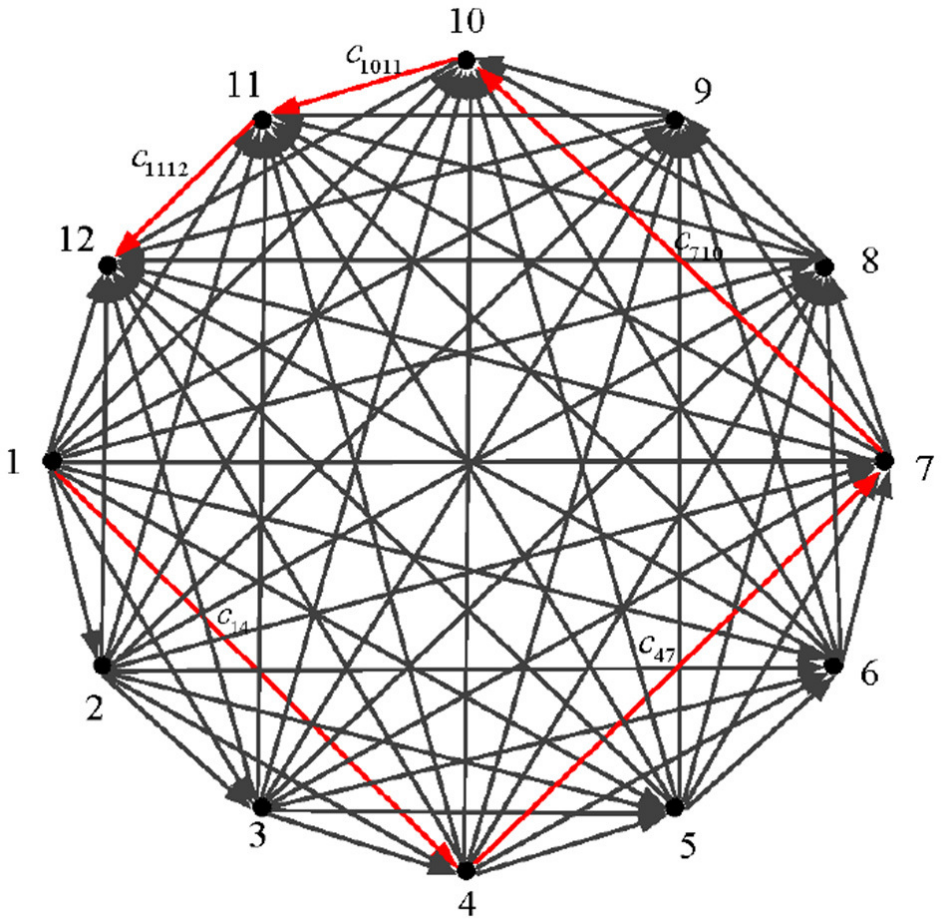
Twelve-node mobile platform path transportation diagram.

To demonstrate the correctness of the energy function mathematical model as well as the validity and real-time capability of the DZNNM, the 12-node path planning problem is further discussed. The transport diagram for the problem is shown in Figure 4. For the sake of comparison, the assumptions of this problem are the same as those of the six-node path planning problem.

For convenience, the initial matrix is defined as follows:

$x\left(0\right)={\left[0,0,\cdots 0\right]}_{144*1}^{⊤}$,

$y\left(0\right)={\left[1,1,\cdots 1\right]}_{12*1}^{⊤}$,

$c={\left[c_{11},c_{12},\cdots c_{112},c_{21},c_{22},\cdots c_{212},\cdots \;,c_{121},\cdots c_{1212}\right]}^{⊤}$.

**Figure 5:**
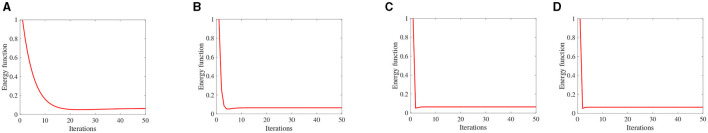
Energy function based on the 12-node primal duality problem, **(A)** ι = 0.1, **(B)** ι = 0.5, **(C)** ι = 0.9, and **(D)** ι = 1.0.

The simulation results of solving the shortest path problem with 12 nodes using the DZNNM are as follows: The simulations show that the path planning problem is increased to 12 nodes, and the DZNNM can effectively solve the discrete TVNOPs, as shown in Table 4. It can be seen from Figure 5 that the addition of path nodes does not influence the convergence rate of the proposed DZNNM.

**Table 4:** Cost coefficients of six-node path planning problems.

	***c*_1_**	***c*_2_**	***c*_3_**	***c*_4_**	***c*_5_**	***c*_6_**	***c*_7_**	***c*_8_**	***c*_9_**	***c*_10_**	***c*_11_**	***c*_12_**
*c*_1_	100	5	4	2	9	8	10	12	25	30	22	23
*c*_2_	100	100	9	10	11	9	9	10	12	15	15	15
*c*_3_	100	100	100	10	11	12	12	8	10	12	15	13
*c*_4_	100	100	100	100	9	10	2	8	12	13	14	13
*c*_5_	100	100	100	100	100	13	12	9	11	12	14	16
*c*_6_	100	100	100	100	100	100	14	13	15	17	18	15
*c*_7_	100	100	100	100	100	100	100	15	14	3	13	19
*c*_8_	100	100	100	100	100	100	100	100	15	17	18	15
*c*_9_	100	100	100	100	100	100	100	100	100	14	15	16
*c*_10_	100	100	100	100	100	100	100	100	100	100	16	3
*c*_11_	100	100	100	100	100	100	100	100	100	100	100	17
*c*_12_	100	100	100	100	100	100	100	100	100	100	100	100

It can reflect the correctness of the path-planning mathematical model as well as the superiority and real-time performance of the DZNNM. In addition, the values in Table 5 show that the energy loss from node 1 to any other node, so it can be concluded that the energy consumption from node 1 to node 4 is the smallest in the path planning process. Figure 5 and Table 5 demonstrate that the DZNNM exhibits convergence performance, 0-stability, and superior capability in handling TVNOPs.

**Table 5:** The energy loss from node 1 to each other.

**|*E*_11_|**	**|*E*_12_|**	**|*E*_13_|**	**|*E*_14_|**	**|*E*_15_|**	**|*E*_16_|**
∞	0.0364	0.0256	0.0113	0.0369	0.0265
|*E*_17_|	|*E*_18_|	|*E*_19_|	|*E*_110_|	|*E*_111_|	|*E*_112_|
0.0217	0.0136	0.0204	0.0645	0.3339	2.1625

Combined with the data in Table 6, the second path consumes the least energy to move from node 4 to node 7. The data in Table 7 show that the optimal choice in the third path is to move from node 7 to node 10, and the energy consumed is 0.0023. Table 8 shows the energy loss of the last two sections of the path. In Table 8, the minimum energy consumption from node 10 to node 11 is reflected by numerical values. Meanwhile, the value of energy consumption from node 11 to target point 12 is given as 1.5876. Combined Table 5 with Table 8, it can be concluded that the motion path in the twelve-node path planning problem is 1 → 4 → 7 → 10 → 11 → 12. The proposed DZNNM is suitable and effective for discrete TVNOPs. In addition, the convergence rate does not decrease with the increase of the nodes in the path-planning problems, and the convergence rate can be accelerated by scaling the design parameters appropriately. These characteristics make the DZNNM suitable for solving large-scale path-planning problems in real-time applications.

**Table 6:** The energy loss from node 4 to each other.

**|*E*_45_|**	**|*E*_46_|**	**|*E*_47_|**	**|*E*_48_|**
0.0121	0.9318	0.0097	0.0125
|*E*_49_|	|*E*_410_|	|*E*_411_|	|*E*_412_|
1.1156	0.1319	0.0218	1.2085

**Table 7:** Energy loss from node 7 to each other.

**|*E*_78_|**	**|*E*_79_|**	**|*E*_710_|**	**|*E*_711_|**	**|*E*_712_|**
0.0218	0.05424	0.0023	0.0719	1.7784

**Table 8:** Energy loss from node 10 to each other.

**|*E*_1011_|**	**|*E*_1012_|**	**|*E*_1112_|**
0.0443	0.2757	1.5876

## 5 Conclusion and future work

A DZNNM is developed and analyzed to handle the shortest path planning problem from a single starting point to a single terminus. For the shortest path planning problem, a discrete nonlinear function related to the energy function is constructed so that the solution of the lowest energy function corresponds to the solution of the shortest path. The shortest path planning problem is transformed into the TVNOPs through strictly mathematical analysis. In addition, the convergence, 0-stability, and theoretical results of the proposed DZNNM are discussed and analyzed, which reflect that the DZNNM can effectively deal with the shortest path-planning problems. Simulation results show that the proposed DZNNM has high precision and real-time performance in dealing with path planning problems. Ultimately, the future research direction is to develop mathematical models under complex conditions and solve multi-starting point and multi-objective point path planning problem.

## Data availability statement

The datasets presented in this study can be found in online repositories. The names of the repository/repositories and accession number(s) can be found in the article/supplementary material.

## Author contributions

FS: Data curation, Formal analysis, Methodology, Writing — original draft, Writing — review & editing. YZ: Data curation, Methodology, Formal analysis, Writing — original draft. CX: Formal analysis, Methodology, Writing — original draft, Writing — review & editing. ZS: Funding acquisition, Methodology, Supervision, Writing — review & editing.
